# Decreased hail size in China since 1980

**DOI:** 10.1038/s41598-017-11395-7

**Published:** 2017-09-07

**Authors:** Xiang Ni, Qinghong Zhang, Chuntao Liu, Xiaofei Li, Tian Zou, Jipei Lin, Hoiio Kong, Zhihua Ren

**Affiliations:** 1 Department of Atmospheric and Oceanic Sciences, School of Physics, Peking University, Beijing, 100871 China; 2 Department of Physical and Environmental Sciences, Texas A&M University at Corpus Christi, Texas, 78412 USA; 3 Information center of Chinese National meteorological Bureau, Beijing, 100081 China

## Abstract

The response of hailstorm intensity to climate variability/change has become a topic of community interest recently. But the lack of persistent and homogenous observations makes it difficult to confidently describe its interannual variations. Hail size, as a common indicator of hailstorm intensity, displays distinct regional variability. Here, for the first time, we show robust evidence of a decrease in hail size using continuous and coherent hail size records from 2,254 manned stations in China since 1980. The stations were categorized based on their elevation: plateaus (above 2000 m), foothills (between 500 and 2000 m), and plains (below 500 m). Compared with 1980–1997, the hail size spectra from 1998 to 2015 all shifted toward smaller sizes significantly in plateaus, foothills, and plains. The proportion of overall hail events with maximum sizes of at least 5 and 20 mm significantly decreased since 1980. Meanwhile, the annual mean size of hail above 10 and 20 mm significantly decreased during the research period, especially after 1990. These changes in the hail size spectra may imply a weakened intensity of hailstorms in China in recent decades.

## Introduction

Variations in hailstorm intensity, have become a controversial topic within the severe weather community in recent years, in addition to the study of hail occurrence changes in different parts of the world^1–7^. The biggest obstacle to our understanding of the interannual and long-term trend of hailstorm intensity is the lack of continuous good quality surface observations. Hailstorm intensity could be measured by various methods, such as the maximum hailstone diameter, number and density of hailstones, fall speed, and swath size^8^. Nevertheless, hail size, usually measured as the maximum diameter of hailstone on the surface ground, is the most common parameter in widely used hailstorm intensity scales^9, 10^. Among various hail observation approaches around the globe, hail size records are documented for different hail ranges, and associated with temporal variations in the recording method and natural variability. Hail sizes from weather stations were reported with minimum size low as 2 mm in four provinces in China^11^, while hailpad station networks have captured a hail size larger than 5 mm in regions of France and Spain^12, 13^. From a 25-yr record of hailpad-observed hail size in France, the number of large hailstones (11–21 mm) was found to have increased together with an increase in the freezing level height^14^. Hail reported by the public is only recorded for hailstones with a size larger than 0.75 inch (19 mm) in the North America region^4, 6^. Among all the hail reports, the fraction of hail greater than 3 inches (7.6 cm) has been declining steadily, while the fraction of hail less than 1.25 inch (3.2 cm) has inflated in the United States (U.S.) since 1955. Although storm chasers and hailstorm-related field programs have improved hail reports quality^6, 15^, variations in the social and non-meteorological factors considered in the studies undertaken with these public hail reports have inevitably led to uncertainties in hail climatology^6^.

The sustained observation of surface weather phenomenon at Chinese weather stations provides an opportunity to monitor the changes in local severe weather phenomenon, including hail, high wind, and lightning^16, 17^. The maximum hail size during each hailstorm has been measured or estimated visually by professionally trained meteorological spotters since the 1950s and has become a routine observation at most manned weather stations in China since 1980. These hail size records are archived at the China Meteorological Administration^16^. According to the specifications for surface meteorological observation, the threshold for hail at a Chinese weather station is solid ice particles in spherical or conical shapes with a size greater than 2 mm^16^, which is smaller than the American Meteorological Society definition of 5 mm. Using hail size records at stations with an elevation below 2000 m from four provincial meteorological bureaus, Xie^11^ showed that there is no significant long-term trends in annual mean hail size among these four regions. In contrast, the highest annual mean hail frequency in China occurs on the Tibetan Plateau^18^, raising the question as to whether the trends seen for lower elevations are also seen for this region. Recently, these manual hail size observations at stations nationwide have been digitized and released after quality control (see data section). The analyses of these data would provide more details of hail size changes in China than Xie’s work and the results can help to the understanding of how hail intensity response to climate variability/changes in China.

## Result

**Figure 1:**
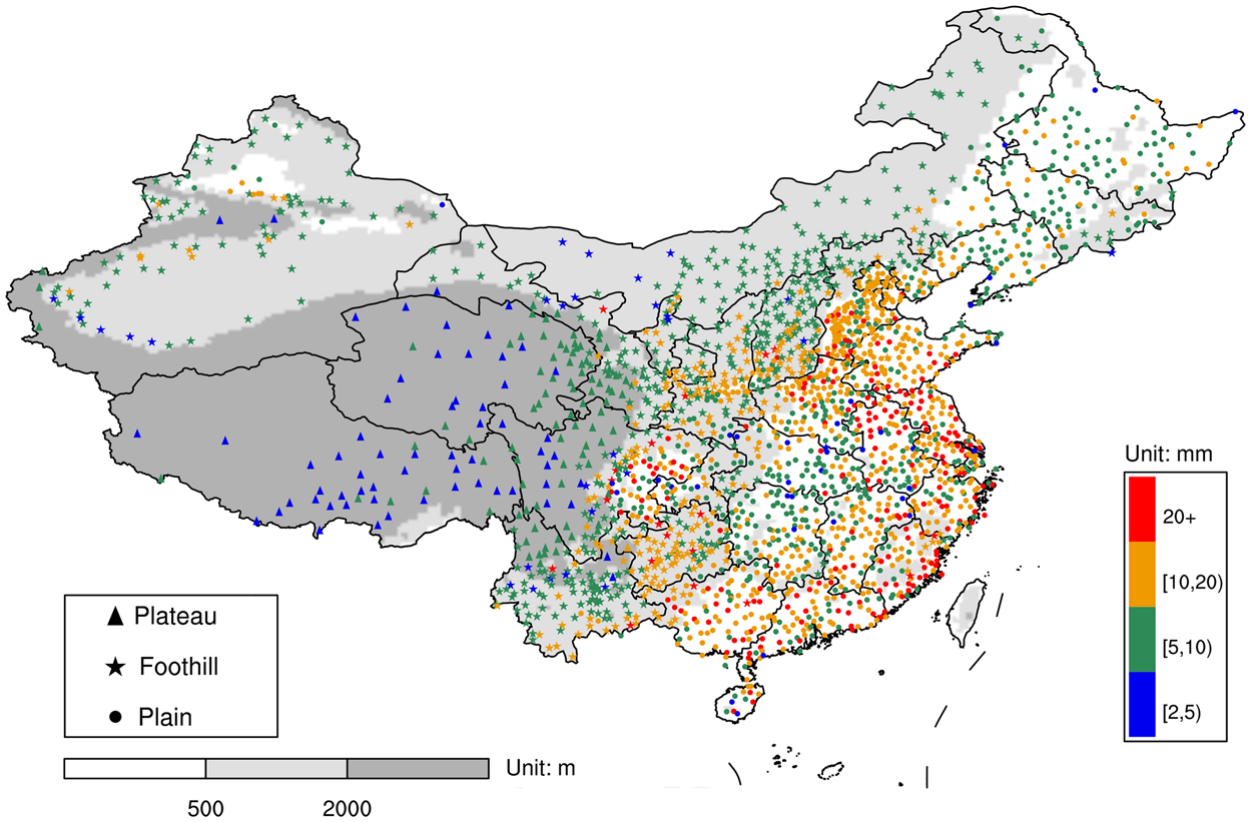
Locations of 2,254 stations and station mean hail size during 1980 and 2015. The stations were divided into three categories according to station elevation: plateaus (>2000 m), foothills (500–2000 m), and plains (<500 m). This figure is generated using NCAR Command Language (NCL)^42^.

Overall, 2,254 stations (Fig. 1) with continuous hail occurrence records and at least one hail size record were divided into three categories based on station elevations: plains (<500 m), foothills (500–2000 m), and plateaus (>2000 m). The plateau stations were mostly located over the Tibetan Plateau. Most plain stations were located in eastern China. Generally, the station mean hail size decreased with an increase in station elevation (Fig. 1 and Supplementary Fig. S2). The stations with a mean hail size greater than 20 mm were mostly located in plain regions, while for the stations in plateau regions, the mean hail sizes were mostly below 10 mm. Satellite observations of convection^19^ and hailstorms in China^20^ also showed similar regional differences to that of storms in plateau or high elevation regions, with lower flash rates and maximum radar reflectivity, which are two common metrics used to indicate convective storm intensity.

**Figure 2:**
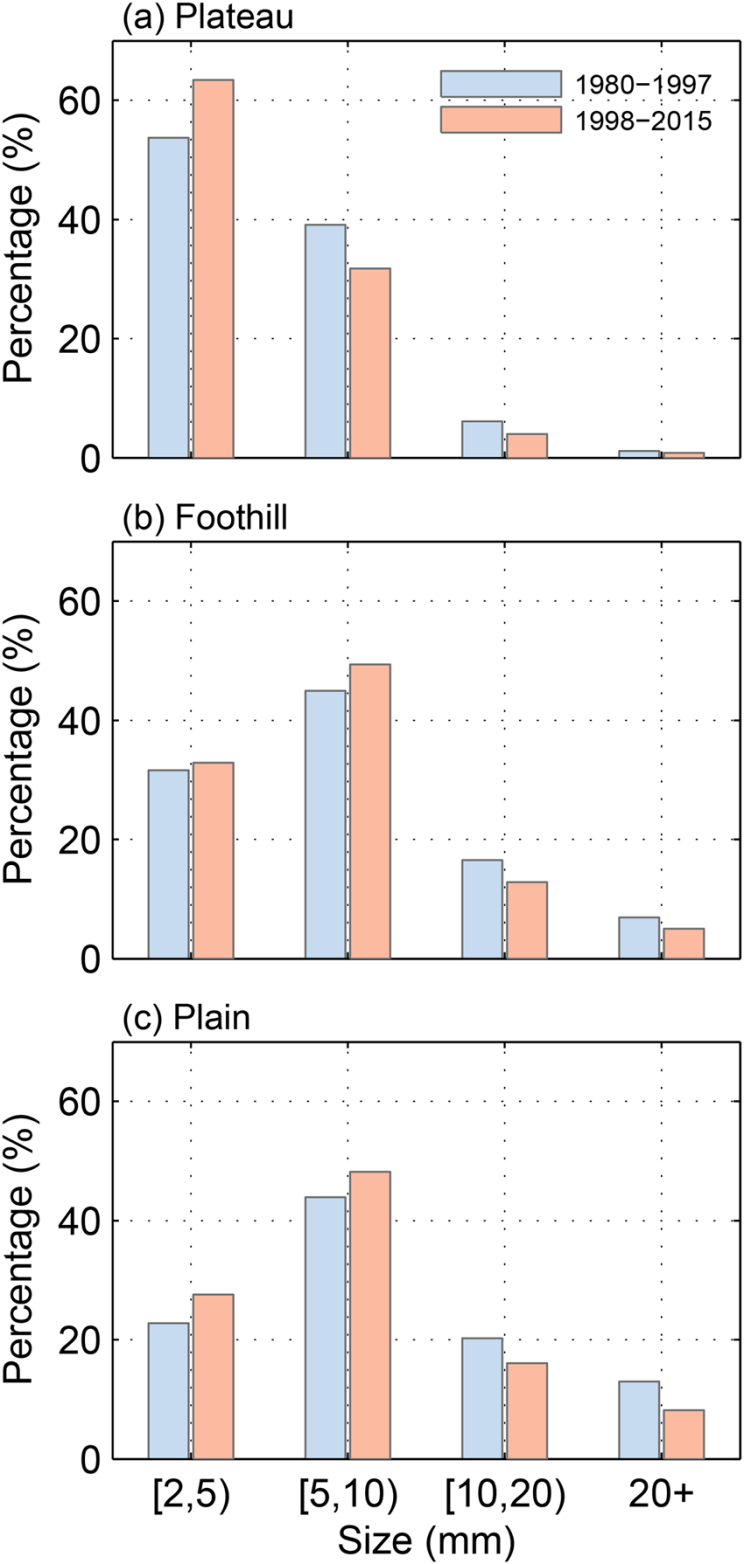
Probability density function (PDF) of hail sizes at stations in different regions from 1980–1997 and 1998–2015. This figure is plotted with MATLAB 2014b.

In the discussion of climate variability, the probability density function (PDF) of meteorological parameters such as temperature, precipitation, and hail size is a good metric of climatology and changes along with climatology variability in different ways. A shift in the PDF of hailstone size in clouds or on the ground in a long-term observation dataset would be a good indicator of changes in hailstorm intensity. According to theories and observations made in clouds and on the ground, the reported PDFs of hail sizes are close to either exponential or gamma distributions^5, 11, 21–23^. The PDFs of hail sizes in three regions are shown in Fig. 2. The hail size PDFs of the plain and foothill regions were close to a gamma distribution, with a maximum probability in the size range between 5–10 mm, which is consistent with the results obtained by Xie^11^. There was a slight difference between the PDFs in plain and foothill regions, with smaller hail in the foothill region. The plateaus has the smallest mean hail size among the three regions, reflecting the climatologically noted lower maximum reflectivity in deep convection^19^. In addition, the PDF of hail size over the plateaus is close to an exponential distribution, which is more consistent with the hail size distribution in cloud^22, 23^. The distinctions among the PDFs in the three regions were consistent with the theoretical calculation^24^ that the thicker the melting depth is, the more the hail size distribution would deviate from an exponential distribution toward a gamma distribution. PDFs with 1-mm-size bins confirmed this finding (Supplementary Fig. S3).

In addition to the different shapes of the PDFs for the three regions, there was a clear shift in the hail sizes during the two periods, as shown in Fig. 2. The PDFs of hail sizes in 1998–2015 shifted to smaller sizes as compared with 1980–1997 over all three regions. In the plains, the proportion of hail smaller than 10 mm changed from 73.91 to 80.50% over the period of 1998–2015. In the plateaus, the probability of small hail (<5 mm) increased from 54.16 to 63.78%. Although the foothills PDF had a similar probability of hail sizes between 2 to 5 mm in both periods, the probability of hail sizes between 5 and 10 mm increased from 47.33 to 51.18% over the second period. On the other hand, accounting for the tail of the distributions, the occurrence of hail larger than 20 mm in the plateaus, foothills, and plains decreased 0.24, 1.91, and 4.81% in the second period, respectively. The distributions are fitted with gamma distribution and applied t-test confirms the significant shifts of the distributions in the second period (See details in the supplementary materials). Overall, as a result of the percentage increase in small sizes and a decrease in the large sizes, the PDFs of hail sizes displayed a robust shift to a smaller size.

**Figure 3:**
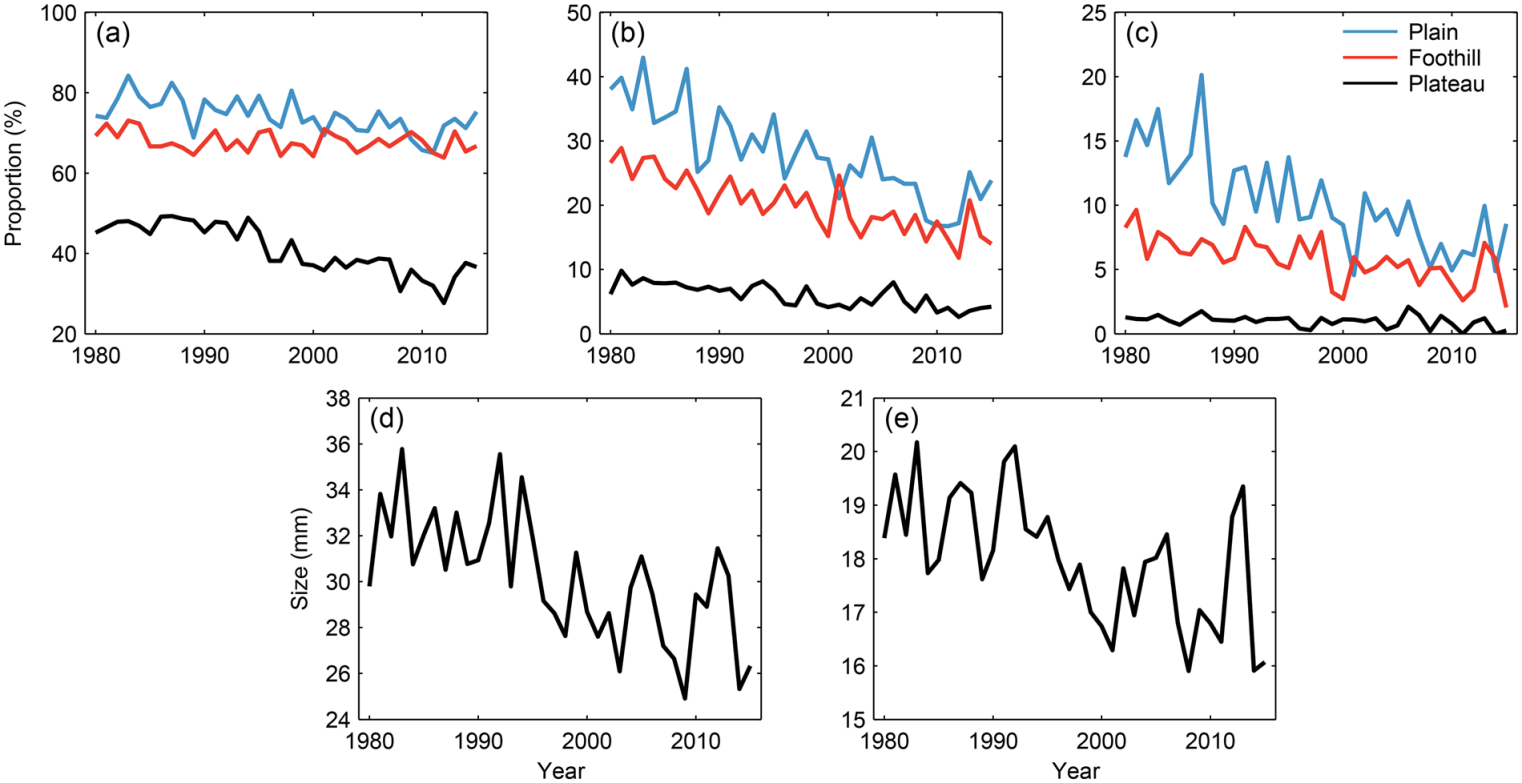
Time series of hail size parameters. (**a**) Interannual variations of the proportion of all hail events with hail size ≥5 mm over plateau, foothill, and plain regions as defined in Fig. 1; (**b**) same as (**a**) but for the proportion of all hail events with hail size ≥10 mm; (**c**) same as (**a**) but for the proportion of all hail events with hail size ≥20 mm; (**d**) annual mean size of hailstones in hail events with hail size ≥20 mm at all 2,254 stations; (**e**) same as (**d**) but for hailstones in hail events with hail size ≥10 mm. This figure is plotted with MATLAB 2014b.

According to data from weather stations, the annual number of hail days and events in China has shown a significant decrease since the 1980s and 1990s in the different regions^1, 7, 17^. To describe the long-term variations of hail size, the proportions of hail events with sizes greater than or equal to 5, 10, and 20 mm in overall hail size records are summarized in Fig. 3. Consistent with the increased probability of small hail sizes in recent years shown in Fig. 2, the proportion of all hail events with sizes greater than or equal to 5, 10, and 20 mm in overall hail records displayed a significant downward trend at a significance level of 0.05 over the study period, although there was a lower significance level of 0.10 for the decline in hail events with hail sizes ≥5 mm in foothill regions. Although the PDFs in Fig. 2 imply similar shifts toward a small hail size range, the interannual variations in the proportion of all hail events with large hailstones displayed distinct regional discrepancies. For example, the proportion of all hail events with large hailstones in plateau regions experienced its largest decrease in the 1990s. In the plains, the proportion of all hail events with hail sizes greater than or equal to 5 and 10 mm displayed a significant downward trend from 1980 onward. The proportion of all hail events with hail sizes ≥20 mm over plain and foothill regions (Fig. 3c) displayed a similar significant downward trend from 1980 onward. In the plains, the proportion of all hail events with hail sizes ≥20 mm in the 2000s dropped to 10% from 18% in 1980s.

Because hail-induced property damage is mainly derived from large hailstones (e.g. 0.75 inch or greater)^3^, the annual mean sizes of hailstones greater than 20 mm at the all 2,254 stations was determined, with the results shown in Fig. 3d. In line with the decreased percentage of 20+ mm hail shown in Fig. 2, the annual mean size of 20+ mm hailstones displayed a decreasing trend of 1.7 mm per decade, significant at 99% level. The decreasing trend in the annual mean size of large hailstones began in the early 1990s, and was similar to the trend in hail frequency observed in North China^7^. The mean sizes of large hailstones in the two periods of 1980–1997 and 1998–2015 were 31.93 and 28.37 mm, respectively. Nationally, there was an average of 96 hail days with hailstones larger than 20 mm per year over the study period, accounting for only 5.37% in the overall hail size reports. The annual mean hail size of hail events with hailstones of at least 10 mm is shown in Fig. 3e. The annual mean hail size has significantly decreased at a rate of 0.7 mm per decade since 1980 at the 99% level. The mean values of annual mean hail sizes in Fig. 3e (≥10 mm) were 18.71 and 17.23 mm in the two periods. The changes in the annual mean size of these large hailstones confirms the surface hail size decrease shown in Fig. 3a–c and the shift in the hail size PDFs shown in Fig. 2.

## Conclusion and Discussion

This study revealed a significant decrease in surface hail size at Chinese weather stations at different elevations over a 36-year period. Station-based hail size records provides a high-quality dataset to study long-term variations of hailstorm intensity. However, the intensity of hail-bearing storm could be defined with different metrics, e.g. hail fall density, swath area, cumulative rainfall, and maximum rain rate^25, 26^. Different metrics may lead to a different conclusion; for example, in model simulations, an increased melting level under a warming climate may lead to decreased surface hail, but a higher risk of storm-induced mountainous floods^26^. From this perspective, the shifts in hail size distributions could not reflect all the changes of hail-bearing storms’ climatology.

Although significant shifts of hail size distribution are presented, the reasons behind these changes are not investigated in this study because of the complexity in environments that regulate hail size variations. The large-scale environmental conditions play a crucial role in the climatology of hailstorm occurrence and intensity and hence dominate the hailstone diameter observed at the surface^27, 28^. Various convective parameters have been shown to be relevant to hailstorm intensity and hailstone diameter, e.g. low-level wind shear, convective available potential energy (CAPE), and freezing level height (FLH). Based on the results of sounding data at weather stations, CAPE are found increase since 1960s in China, along with the decrease of vertical wind shear^1, 17^. In the context of climate warming, climate models projected increase of CAPE and decrease in wind shear, resulting in increase of thunderstorm days^29^. Nevertheless, the intensity of a given significant hailstorm is much more dependent on shear than on thermodynamic parameters^28^. On the other hand, the FLH influences hail size on the ground through changes in the melting layer thickness and adjustments in the hail growth zone^11, 23, 26^. These mechanisms with various consequences make the understanding of hail size distribution changes more complex from thermodynamic and microphysical views.

In addition to these dynamic and thermodynamic factors, cloud microphysical processes could also influence the development of hailstorms. By providing artificial ice nuclei, weather modification projects to suppress hailstorms in China have been conducted for several decades^30^. However, the effects of these hail suppression projects have not been fully evaluated^31^. In some hail suppression programs, an increase in the number of hailstorms has been reported^31, 32^. In addition, the persistence of aerosol pollution is considered to be a cause of convection invigoration, which enhances the growth of large hail^33, 34^. To the contrary, the observed downward trend in thunderstorm activity has also been attributed to the suppression of heavy pollution^35^. Similarly, the cloud condensation nuclei concentration is found to have non-linear effects on the microphysical processes of hail growth^36^. Overall, these unresolved complex interactions between various environmental factors and hailstorms have made it difficult to attribute the decreased hail size in China to any specific factor.

At this point, the decrease of hail occurrence and hail size in China are two corroborated characteristics of hailstorms. However, the hailstorm occurrence and hail intensity are independent from each other from climate perspective. Hail occurrence indicates how often the hailstorm occur and hailstorm intensity measured by hail size means how intense the storm would be for a given hailstorm. How the environments impacts the two characteristics would be considered separately^37^. Although areal variation of favorable environments for storm development and hail occurrence have been widely documented^38, 39^, the connections between inter-annual variations in hail size and local environment are still not fully understood. Further studies of the association of regional severe convective weather with the microphysical effects of aerosols and large-scale circulations, such as the El Niño/Southern Oscillation, Arctic Oscillation, and Madden–Julian Oscillation^40, 41^, are required to improve our understanding of hailstorm intensity variations.

### Data

Hail reports for storms with hailstones of at least 2 mm in size from 2,477 stations in China were compiled with the help and authorization of the Chinese National Meteorological Information Center (CNMIC) in China. The raw dataset is not allowed to redistribute ourselves per CNMIC data use policy, but is accessible upon request. The resulting dataset included occurrence flag, maximum size, starting time, and ending time of hail that occur at the weather station and also of some hail reports in same administrative region. For each station, if there was at least one hail report in one day, the day was defined as a hail day. At the 2,477 stations, there were 81,507 hail days between 1980 and 2015. To ensure a relatively large and continuous dataset, if hail occurrence observations were missing for a month or more in a station during the 36 years, this station was regarded as invalid station. Finally, 2,254 stations with continue observations had been chosen in the dataset from 1980 to 2015. The annual proportion of all hail days with size record are shown in Supplementary Fig. S1. For most hail days in the study period the size of hailstones was recorded. The proportion of hail days in which there was record of the size of hailstones was less than 80% in only 5 of the 36 years period. In this study, we used records for all hail sizes to study the variability of surface hailstone size. Taking the factor of station elevation into account, the 2,254 stations were divided into three categories: plateaus (above 2000 m with 157 stations), foothills (between 500 m and 2000 m with 712 stations), and plains (below 500 m with 1,385 stations).

## Electronic supplementary material

Supplementary information
