# Supplementary material for: Decreased hail size in China since 1980
Source: Sci Rep. 2017 Sep 7;7:10913. doi: 10.1038/s41598-017-11395-7 (PMC5589923; doi:10.1038/s41598-017-11395-7)
Supplement: Supplementary file 1 — Supplementary information [file 41598_2017_11395_MOESM1_ESM.pdf]

Supplementary Information of

Decreased hail size in China since 1980

Authors: Xiang Ni, Qinghong Zhang, Chuntao Liu, Xiaofei Li, Tian Zou, Jipei Lin, Hoiio Kong, and Zhihua Ren

Sueppplementray Table

Supplementary Table S1. Statistical parameters for the gamma distribution fitting and t-test at 0.01 significance level. The t-test is done with function of MATLAB (2014b).

	Tibet		Foothill		Plains	
	1980-1997	1998-2015	1980-1997	1998-2015	1980-1997	1998-2015
Number	17509	9886	14425	6922	10726	5347
a*	3.0938	3.1279	2.2673	2.6260	2.1388	2.3139
b	1.6542	1.4519	3.4134	2.7190	4.3694	3.4969
Mean	5.1179	4.5413	7.7391	7.1402	9.3455	8.0915
Variance	8.4662	6.5933	26.4166	19.4142	40.8344	28.2948
t-test	17.0000		8.7963		13.1465	

* The gamma distribution is assumed as $\mathrm{f(x)} = \frac{x^{a-1}\exp(-x/b)}{b^{a}\Gamma(a)}$

Supplementary figures

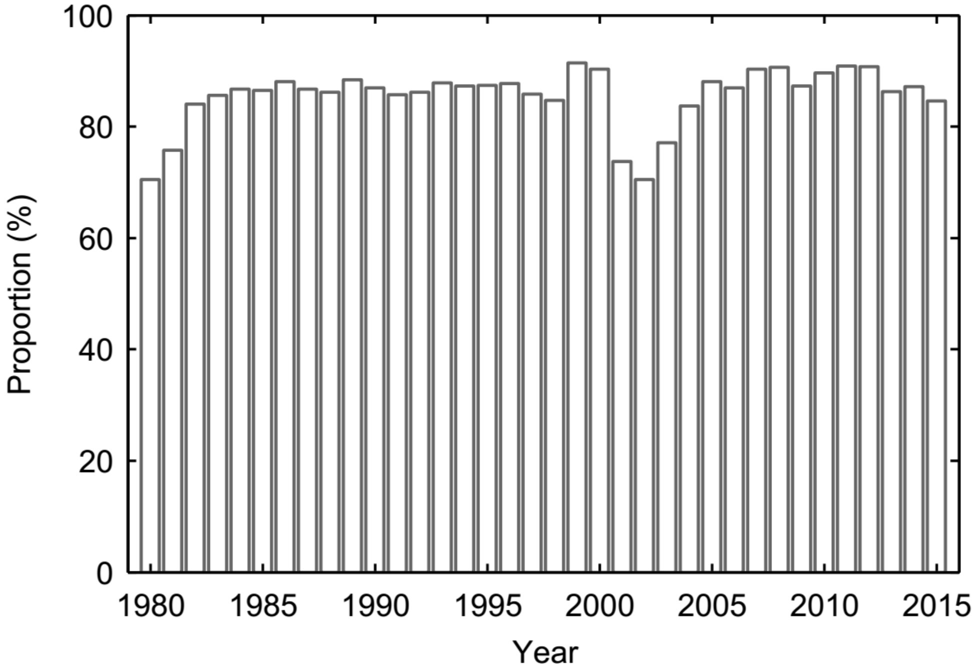

Supplementary Fig. S1 Proportion of hail days for which hail size was recorded at 2,254 stations in China from 1980 to 2015. This figure is plotted with MATLAB 2014b.

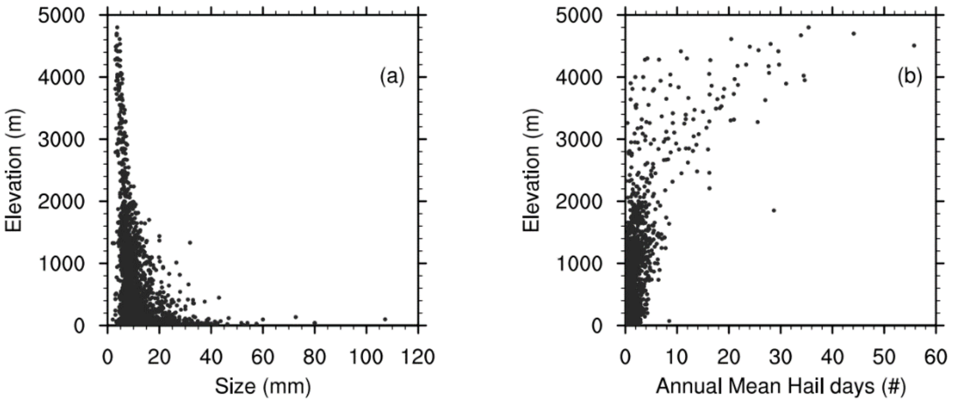

Supplementary Fig. S2 Station elevation versus mean hail size (a) and annual mean hail days (b) at 2,254 stations from 1980–2015. This figure is generated using NCAR Command Language (NCL).

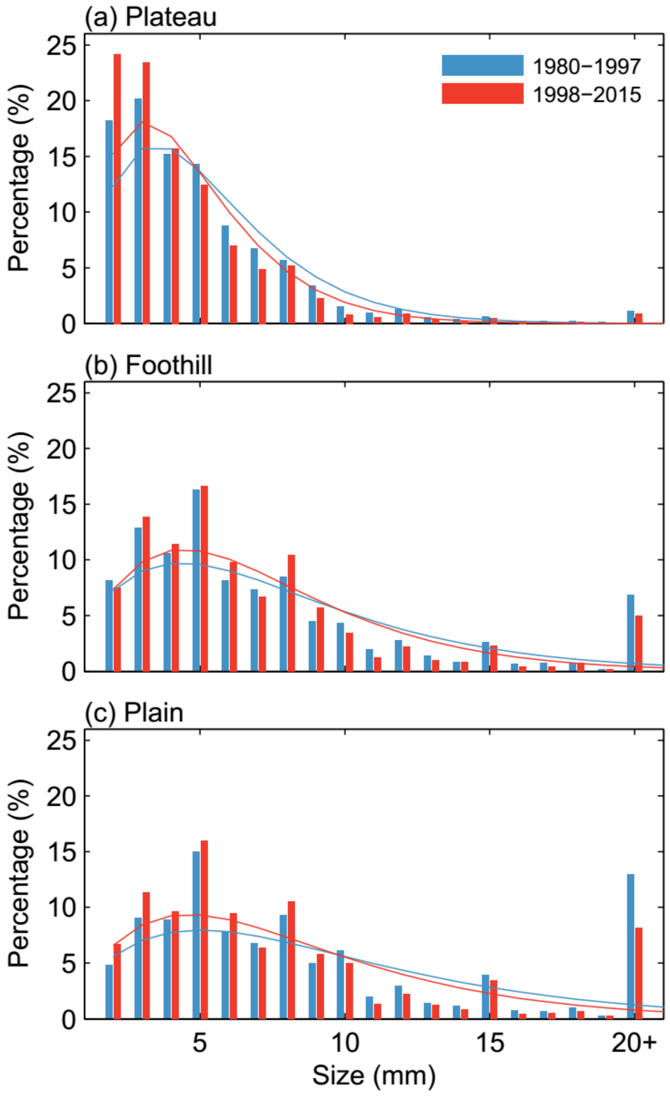

Supplementary Fig. S3 Same as Fig 2, but with 1-mm-size intervals. The solid lines are fitted gamma distribution. Note that the percentages of hail (≥20 mm) are summed up in the plot, but separated in the gamma distribution fitting. The detailed statistical parameters are shown in Tabel1. This figure is plotted with MATLAB 2014b.

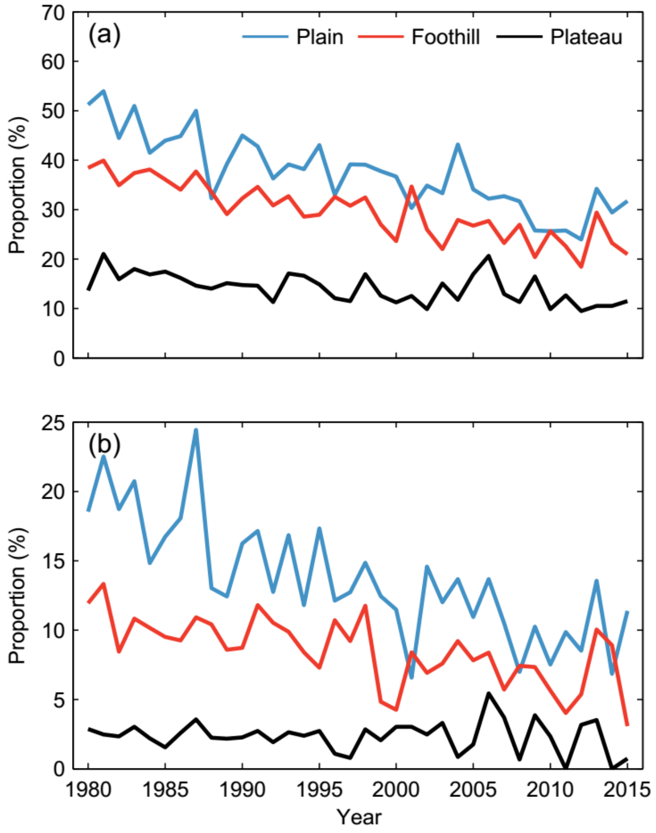

Supplementary Fig. S4 Proportion of (a) number of hail events with hail size ≥ 10 mm and (b) number of hail events with hail size ≥ 20 mm to the number of hail events with hail size ≥ 5 mm from 1980–2015. This figure is plotted with MATLAB 2014b.
